# Relationship Between Parenting Styles and Personality in Older Spanish Adolescents

**DOI:** 10.3390/bs15030339

**Published:** 2025-03-10

**Authors:** Celia Cuadri, Joan García-Perales, Isabel Martínez, Feliciano Henriques Veiga

**Affiliations:** 1 Department of Psychology, University of Castilla-La Mancha, Avda. Los Alfares 44, 16071 Cuenca, Spain; celia.cuadri@alu.uclm.es; 2 Department of Psychology, Selart European University, Avda. Aragón, 10, 46021 Valencia, Spain; joangp@uv.es; 3 Instituto de Educação, Universidade de Lisboa, Alameda da Universidade, 1649-013 Lisboa, Portugal; fhveiga@ie.ulisboa.pt

**Keywords:** parenting styles, personality, extraversion, agreeableness, conscientiousness, emotional stability, intellect, self-esteem, life satisfaction, adolescents

## Abstract

Adolescence is a critical period for identity formation and psychological adjustment, where parenting styles play a fundamental role in shaping socialization and emotional development. The present study analyzed the relationships of parenting styles with the Big Five personality traits (extraversion, agreeableness, conscientiousness, emotional stability, and intellect), self-esteem, and life satisfaction in older Spanish adolescents. A sample of 366 Spanish university students (69.1% girls and 30.9% boys) aged 18 and 19 years completed measures assessing parenting styles, personality traits, self-esteem, and life satisfaction. Data analysis involved a multivariate approach, considering the four parenting styles as independent variables. The results of the MANOVA test indicate that adolescents raised with indulgent and authoritative parenting exhibit significantly higher levels of extraversion, agreeableness, conscientiousness, emotional stability, self-esteem, and life satisfaction compared to those raised with neglectful or authoritarian parenting. These findings highlight the influence of parenting styles not only on adolescent well-being, but also on personality development.

## 1. Introduction

Adolescence is a critical developmental period characterized by identity formation, value consolidation, and increasing social interactions (Metwally, 2018). This phase is marked by rapid biological changes, including growth in height and weight, as well as the development of primary and secondary sexual characteristics (Lamborn et al., 1991; Steinberg et al., 1992, 1994). These transformations, combined with increased social interactions and cognitive advancements, make adolescents particularly sensitive to their environment (Van Heel et al., 2019). Within this context, family dynamics play a crucial role in shaping their emotional and behavioral responses, contributing to the formation of personality traits and social adaptation (Calders et al., 2019). During this stage, parents play a fundamental role in children’s socialization, influencing their emotional and psychological adjustment (Delgado et al., 2022). As the primary agents of socialization, parents introduce children to social norms, expectations, and cognitive patterns that guide their development. Parenting styles are characterized by consistent patterns of parental responses to children’s behaviors across various everyday situations, reflecting their approach to guidance and socialization (Baumrind, 1991; Darling & Steinberg, 1993). These parental styles create the broader family context in which adolescents grow, influencing key developmental outcomes such as social maturity and adherence to social norms (Flores Talavera, 2021).

A widely accepted framework for understanding parenting behavior is based on two primary dimensions: strictness and warmth (Maccoby & Martin, 1983; Sun et al., 2023). Strictness represents the degree of control or authority that parents exercise over their children. Warmth refers to the sensitivity, affection, support, and acceptance that parents exercise with their children. Based on these two dimensions, four parenting styles have been identified: authoritative (high strictness, high warmth), authoritarian (high strictness, low warmth), indulgent (low strictness, high warmth), and neglectful (low strictness, low warmth) (Martínez et al., 2019). Recent research suggests that authoritative and indulgent parenting styles, both characterized by high warmth and reasoning practices, are more beneficial for adolescent development than authoritarian and neglectful styles, which rely less on emotional support and reasoning (Calafat et al., 2014; Cerezo et al., 2011; Di Maggio & Zappulla, 2014; Serna et al., 2023).

Beyond parenting, adolescence is also a crucial period for personality development. As individuals transition from childhood to adulthood, they experience significant physical, cognitive, and social changes that influence the formation and stability of personality traits (Van Heel et al., 2019). Additionally, adolescents challenge societal norms and values while seeking greater independence, further shaping their personality development and long-term psychological well-being (Van Heel et al., 2019). Personality is commonly understood as a set of psychological traits that shape an individual’s thoughts, emotions, and behaviors (Cervone & Pervin, 2022). Individuals’ personalities develop through the interplay of temperament, character, and environmental influences and play a vital role in self-awareness, social integration, and personal growth (Metwally, 2018). Furthermore, personality traits established in adolescence often persist into adulthood, impacting long-term social and psychological well-being (Metwally, 2018; Saiz et al., 2011).

A widely used framework for describing personality is the Big Five Model, which identifies five major dimensions: (1) extraversion, which includes the ability to stand out, assert oneself, and influence others, and enthusiasm and energy; (2) agreeableness, which comprises the ability to listen to others and cooperate, and politeness; (3) conscientiousness, which includes persistence, perseverance, meticulousness, and organization; (4) emotional stability (or the inverse trait, neuroticism), which encompasses control of behavior and emotions; and (5) intellect (also called openness or imagination), which covers different values or ways of life (openness to experience) and interest in acquiring new knowledge and staying informed (openness to culture) (McCrae & Costa, 1997).

Another key psychological factor during adolescence is self-esteem, which significantly influences emotional and social well-being (Álvarez Aguirre et al., 2010). Self-esteem refers to the set of thoughts and feelings that a person has about him/herself and the degree of personal approval or rejection (Rosenberg, 1965). It develops through interactions with the environment and is shaped by external reinforcement and significant relationships, affecting cognitive, emotional, and behavioral functioning (Shavelson et al., 1976).

Additionally, life satisfaction has gained research interest as a critical aspect of adolescent well-being (Diener et al., 1999). Life satisfaction refers to an individual’s overall assessment of the quality of his or her life, including their moods and the immediate effects of life events (Diener et al., 1985). Given that adolescents face numerous life transitions, particularly related to education and future planning, maintaining high life satisfaction is essential for their long-term development (Marcionetti & Rossier, 2016). Both self-esteem and life satisfaction have been related to a wide variety of positive psychological and behavioral outcomes, such as psychological adjustment, positive emotion, school engagement, bullying and cyberbullying or Internet addiction, health, effective interpersonal relationships, and school dropout (Bahrainian et al., 2014; Leary & MacDonald, 2003; Martínez et al., 2021; Veenhoven, 1988).

Extensive research has explored the relationship between parenting styles and adolescent adjustment, showing connections with variables such as self-esteem, emotional instability and emotional unresponsiveness, school performance, use of learning strategies, and substance use or behavioral problems (Calafat et al., 2014; Fuentes et al., 2015; Martínez et al., 2013). Initial studies conducted primarily in the United States with European–American families found that the authoritative parenting style was associated with numerous positive outcomes, such as lower substance use, healthier lifestyle choices, and greater psychosocial competence (García et al., 2019; Steinberg et al., 1994; Jackson et al., 1998; Kremers et al., 2003; Radziszewska et al., 1996). In those early studies, adolescents from authoritative families tended to exhibit better psychological and behavioral adjustment than those from authoritarian, indulgent, or neglectful households (e.g., Lamborn et al., 1991; Maccoby & Martin, 1983).

However, emerging research conducted in Spain (Cerezo et al., 2011) and other European countries, such as Portugal (Rodrigues et al., 2013), Italy (Di Maggio & Zappulla, 2014), Germany (Wolfradt et al., 2003), Turkey (Turkel & Tezer, 2008), and Norway (Lund & Scheffels, 2018), and also in some Latin American countries, such as Mexico (Villalobos et al., 2004) and Brazil (Martínez et al., 2019), has consistently demonstrated that warmth-based parenting practices, characteristic of both authoritative and indulgent styles, are associated with the highest levels of adolescent adjustment. Authoritative and indulgent parenting styles have been linked to lower psychological distress in emerging adulthood (Parra et al., 2019), reduced antisocial behaviors (Martínez et al., 2013), increased self-esteem and internalization of social values (Martínez et al., 2020), and overall better psychosocial adjustment (García et al., 2019). Furthermore, in Spain, indulgent parenting has also been identified as a protective factor against sexist prejudice, violence toward parents, substance use, and bullying victimization (Gámez et al., 2012; Garaigordobil & Aliri, 2012; Martínez et al., 2019).

Although previous research has extensively examined the relationship between parenting and adolescent adjustment, studies have primarily focused on outcomes such as self-esteem, academic performance, and substance use (Martínez et al., 2024, 2020). In contrast, the connection between parenting styles and adolescent personality traits remains quite underexplored. Understanding these dynamics is essential for a comprehensive analysis of adolescent personality traits and their long-term implications. Furthermore, this knowledge can provide valuable insights into educational strategies, mental health interventions, and policies aimed at fostering well-adjusted individuals in society.

While some studies suggest that high parental control is associated with lower scores for the Big Five personality traits, whereas warmth-based practices correlate with higher scores (Lianos, 2015; Tehrani et al., 2024), research has yet to systematically analyze the differential impact of all four parenting styles (authoritarian, authoritative, indulgent, and neglectful) on adolescent personality.

The present study aims to examine the relationships between parenting styles (authoritative, authoritarian, indulgent, and neglectful) and personality traits in older Spanish adolescents. Additionally, we will explore how parenting styles relate to self-esteem and life satisfaction. Based on recent research (Calafat et al., 2014; Martínez et al., 2019, 2020; Serna et al., 2023), we hypothesize that authoritative and indulgent parenting styles, characterized by high warmth, will be associated with higher scores for the Big Five personality traits, self-esteem, and life satisfaction compared to authoritarian and neglectful parenting styles. Furthermore, we do not expect significant gender-based interactions in these associations (Martínez et al., 2007, 2013, 2021).

## 2. Materials and Methods

### 2.1. Participants

The study sample consisted of 366 university students from three universities in the eastern region of Spain. All of them were in the first year of university; 113 (30.9%) were male and 253 (69.1%) female. The participants were aged between 18 and 19 years old (M = 18.61, SD = 0.48). To ensure a statistical power of 95% (1 − β = 0.95), an a priori calculation was conducted using G*Power 3.1.9.7 (Faul et al., 2009) to determine the minimum required sample size. The analysis set the Type I error rate at the conventional threshold (*α* = 0.05) and considered a medium effect size (*f* = 0.21) (Cohen, 1977) in the F-test between the four parenting styles. The findings indicated that at least 350 participants would be needed for the study.

### 2.2. Procedure

From a comprehensive list of the first-year courses at the universities of the region, 10 classes were randomly selected through simple random sampling. According to Kalton (1983), when groups (e.g., educational institutions) are randomly selected, the individuals within those groups (e.g., students) will be similar to what a random system would provide. A professor from each selected class was then contacted to facilitate the online administration of the survey. Student participation was entirely voluntary, with no compensation provided, and all responses were collected anonymously. Participants were thoroughly informed about the procedure for completing the instruments, as well as the conditions regarding the anonymity and confidentiality of the survey, ensuring that they could provide honest and sincere responses. All the respondents completed a series of standardized psychological scales. Subscale and total scores were computed as the mean of the corresponding items. Those items that required reverse scoring were appropriately recoded before calculating the total scores. Given that the survey was administered online, the proportion of missing data was minimal, accounting for less than 5%. Finally, the study was conducted following the ethical principles outlined in the Declaration of Helsinki.

### 2.3. Measures

The warmth and strictness dimensions were measured with the Warmth/Affection Scale (Rohner, 2005) and Parental Control Scale (Rohner & Khaleque, 2003), both integrated in the Parental Acceptance-Rejection/Control Questionnaire. The Warmth/Affection Scale is made up of 20 items that evaluate the warmth dimension, where the adolescents rate the frequency with which their parents are affectionate, responsive, and involved with them (e.g., “They make me proud when I do things right”). The Parental Control Scale is composed of 13 items that evaluate the strictness dimension, where the adolescents rate the frequency with which they perceive that their parents monitor them in an imposing, firm, and demanding way (e.g., “They tell me exactly how I have to do things”). Both scales have a response scale ranging from 1 (“rarely true”) to 4 (“almost always true”), so that high scores indicate a high degree of warmth and strictness. The Cronbach’s alpha values obtained in this study for both the dimensions were, respectively, 0.95 and 0.84.

Following the examples of Lamborn et al. (1991) and Steinberg et al. (1994), families were labeled into four types of parenting styles. Authoritative families were those who scored above the 50th percentile on both the warmth and strictness dimensions, whereas neglectful families were below the 50th percentile on both dimensions. Indulgent families were above the 50th percentile on warmth and below the 50th percentile on strictness. Authoritarian families were above the 50th percentile on strictness and below the 50th percentile on warmth.

Personality traits were captured with the Mini-IPIP Scale (Donnellan et al., 2006), which is the abbreviated version of the IPIP (International Personality Item Pool) and has five factorial dimensions (Goldberg, 1992). This scale was translated into Spanish by Martínez-Molina and Arias (2018), and found reliabilities between 0.71 and 0.79 for the five subscales. The Likert-type scale has five points ranging from 1 (strongly disagree) to 5 (strongly agree). Respondents responded to the 20 items (four per dimension): extraversion (BFE) (e.g., “ I am the life of the party”), agreeableness (BFA) (e.g., “I sympathize with others’ feelings”), conscientiousness (BFC) (e.g., “I get chores done right away”), emotional stability (BFS) (originally formulated in the inverse sense of neuroticism) (e.g., “I have frequent mood swings”), and intellect (BFI) (e.g., “I have a vivid imagination”). In this study, the reliability of the Mini-IPIP showed a Cronbach’s alpha of 0.91, and for each factor, the reliability was 0.72 (BFE), 0.78 (BFA), 0.75 (BFC), BFS (0.79), and BFI (0.81).

Self-esteem was measured using the Rosenberg (1965) Self-Esteem Scale, translated into Spanish by Atienza et al. (2000) and reporting a reliability of 0.87. Respondents answered the 10-item questionnaire, in which they were asked to rate items on a four-point Likert scale from 1 to 4 (Strongly disagree, Disagree, Agree, Strongly agree) (e.g., “Overall, I am satisfied with myself.”). This scale has been validated in numerous countries (e.g., Schmitt & Allik, 2005), and its original reliability was 0.77 for this study; the reliability of the Self-Esteem Scale showed a Cronbach’s alpha of 0.86.

Satisfaction with life was captured with the Satisfaction with Life Scale (SWLS) developed by Diener et al. (1985), which was translated into Spanish by Atienza et al. (2003) and had a reliability of 0.91. It is a one-dimensional instrument consisting of five items that evaluates global cognitive judgment of satisfaction with one’s life. The Likert-type scale has four points rated 1 (Strongly disagree) to 4 (Strongly agree). Higher scores reflect greater satisfaction (e.g., “If I could live my life again, I would repeat it just as it has been”). In this study, the reliability of the Satisfaction with Life Scale showed a Cronbach’s alpha of 0.87.

### 2.4. Design and Statistical Analysis

A MANOVA factorial test was applied (4 × 2) for the set of five personality traits (extraversion, agreeableness, conscientiousness, emotional stability, and intellect), self-esteem, and satisfaction with life, and considering socialization styles (authoritative, indulgent, authoritarian, and neglectful) and sex (male or female) as independent variables to test for possible interaction effects. The dataset meets the necessary statistical criteria for MANOVA: independence of observations, multivariate normality, homogeneity of variances, and linearity. Univariate *F* tests were then carried out to examine the differences in the criteria, and Bonferroni’s post hoc test was applied. Statistical analyses were performed using the software package SPSS 28.0.

## 3. Results

### 3.1. Parenting Style Groups

Adolescents were registered into one of four parenting style groups (indulgent, authoritative, authoritarian, or neglectful) (Table 1). Indulgent, 107 (29.2%), with high warmth, M = 3.75, SD = 0.14, and low strictness, M = 1.32, SD = 0.15; authoritative, 59 (16.1%), with high warmth, M = 3.75, SD = 0.14, and high strictness, M = 2.56, SD = 0.432; authoritarian, 141 (38.51%), with low warmth, M = 2.25, SD = 0.54, and high strictness, M = 2.76, SD = 0.47; and neglectful, 59 (16.1%), with low warmth, M = 2.28, SD = 0.84, and low strictness, M = 2.07, SD = 0.16. Additional analyses also showed that both parental dimensions, warmth and strictness, following the orthogonality assumption, were modestly correlated: *r* = −0.381, *R*^2^ = 0.15, *p* < 0.001.

### 3.2. Main Effects

As was hypothesized, the MANOVA test showed a significant main effect for parenting style: Λ = 0.697, *F*(21, 1011.30) = 6.46, *p* < 0.001 (Table 2). Additionally, the main effects for sex, Λ = 0.912, *F*(7, 352) = 64.48, and *p* < 0.001, were significant (Table 2). No interaction effects between parenting styles and sex were found: Λ = 0.912, *F*(21, 1011.30) = 1.56, *p* > 0.05 (Table 2).

### 3.3. Parenting Style Effects

Follow-up univariate analyses (ANOVAs) indicated statistically significant effects for parenting in four of the five dimensions of personality (extraversion, agreeableness, conscientiousness, and emotional stability), self-esteem, and satisfaction with life.

As shown in Table 3, regarding personality, significant differences were found for extraversion (*F*(3, 358) = 3.03, *p* < 0.05), showing higher scores for indulgent than authoritarian parenting. The agreeableness personality trait shows significant differences (*F*(3, 358) = 16.79, *p* < 0.001). Indulgent parenting shows higher scores than authoritative and authoritarian parenting. Furthermore, neglectful parenting shows higher scores than authoritarian parenting in agreeableness. In the conscientiousness personality trait (*F*(3, 358) = 3.48, *p* < 0.05), the students show higher scores with indulgent than with authoritarian parenting. Regarding the emotional stability personality trait (*F*(3, 358) = 9.08, *p* < 0.001), students from authoritarian or neglectful families show lower scores than students from indulgent or authoritative families. Finally, the intellect personality trait (*F*(3, 358) = 1.54, *p* > 0.05) does not show significant differences between parenting styles.

Regarding self-esteem and satisfaction with life, students who qualified their parents as authoritarian or neglectful show lower scores for self-esteem *(F*(3, 358) = 20.67, *p* < 0.001) and for satisfaction with life (*F*(3, 358) = 26.25, *p* < 0.001) than students from indulgent and authoritative families. No statistically significant differences were found between indulgent and authoritative styles nor between authoritarian and neglectful styles.

### 3.4. Univariate Effects of Sex

Follow-up univariate analyses (ANOVAs) for sex indicated statistically significant effects for two personality traits. Girls showed higher levels of agreeableness (*F*(1, 356) = 11.43, *p* < 0.001) than boys, whereas boys showed higher levels of emotional stability (*F*(1, 356) = 16.87, *p* < 0.001) than girls (Table 4). No significant differences were found in the other personality traits, neither in self-esteem nor in satisfaction with life.

## 4. Discussion

Overall, the findings of this study support the initial hypotheses; authoritative and indulgent parenting styles are associated with higher scores for the Big Five personality traits, self-esteem, and life satisfaction among older Spanish adolescents.

First, the results show that parental socialization styles are significantly related to four of the five personality dimensions—extraversion, agreeableness, conscientiousness, responsibility, and emotional stability. Adolescents raised in indulgent and authoritative households tend to exhibit higher levels of these traits compared to those raised in authoritarian or neglectful homes, with the authoritarian style consistently showing the lowest scores. These results reinforce previous research suggesting that strict parental control is related to low scores on children’s personality traits (Asanjarani et al., 2022). Previous studies showed that strict control is associated with being less extroverted, less kind, less responsible, and with adolescents’ little emotional stability (Ayoub et al., 2021). The results also align with recent studies conducted in European countries, which highlight the positive effects of indulgent and authoritative parenting on different variables of adolescent adjustment (Calafat et al., 2014; Cerezo et al., 2011; Di Maggio & Zappulla, 2014; Flores Talavera, 2021; García et al., 2019; Lund & Scheffels, 2018; Martínez et al., 2021). It should be noted that in our study, openness to experience was not significantly linked to parenting style. This raises a question about whether certain personality dimensions might be more or less susceptible to the influences of parenting, suggesting that the relationship between parenting and personality may be more complex than previously assumed.

Second, this study confirms that self-esteem and life satisfaction are significantly higher among adolescents raised under indulgent and authoritative parenting styles compared to those raised in authoritarian or neglectful households. This supports previous findings, which consistently show that parenting characterized by warmth and emotional support is associated with positive psychological well-being, including higher self-esteem and greater life satisfaction (e.g., Martínez et al., 2007; Xie et al., 2016).

Additionally, although not the primary focus of this study, sex differences were observed in two personality traits: agreeableness and emotional stability. In line with prior research (Costa et al., 2001; Weisberg et al., 2011), girls scored higher in agreeableness, while boys scored higher in emotional stability. This finding underscores the role of biological and social influences in shaping personality traits during adolescence.

This study expands upon previous research by showing the significant role of warmth-based parenting (indulgent and authoritative) in shaping not only adolescent adjustment, but also core personality traits. While much of the literature has focused on the effects of parenting styles on behavioral outcomes and psychological well-being, this study highlights how parental warmth contributes to long-term personality development. These findings emphasize the importance of positive parenting practices in fostering well-adjusted individuals who are socially engaged, emotionally stable, and psychologically resilient.

Finally, this research presents some limitations. First, its cross-sectional design prevents any definitive conclusions about causality or the directionality of the observed relationships. Second, parenting styles were assessed through self-reports from university students, which can generate social desirability. While previous studies suggest that adolescents’ perceptions of parenting styles are reliable and consistent across different data collection methods (Baumrind, 1991; Steinberg et al., 1994), self-reports remain susceptible to social desirability bias. Third, the sample consisted exclusively of university students, which limits the generalizability of the findings to the broader adolescent population.

In conclusion, this study reinforces the importance of warmth-based parenting (both authoritative and indulgent) in fostering positive adolescent development. It expands upon previous research by showing that parental styles are not merely associated with behavioral outcomes, but are also instrumental in shaping core personality traits, extending the benefits of indulgent and authoritative parental styles to personality development. This study further underscores that the benefits traditionally attributed to authoritative and indulgent parenting styles extend beyond externalized behaviors to deeper, intrinsic aspects of personality formation. These findings underscore the need for parenting interventions that promote warmth and support as key factors in raising well-adjusted individuals. Further research should explore these dynamics across different cultural contexts and through longitudinal designs to better understand how early parental influences shape personality across people’s lifespans.

## Figures and Tables

**Table 1:** Number of cases in parenting style groups, and mean scores and standard deviations on measures of parental dimensions.

	Total	Indulgent	Authoritative	Authoritarian	Neglectful
Frequency	366	107	59	141	59
Percent	100	29.20	16.10	38.5	16.1
Warmth					
*Mean*	2.94	3.76	3.75	2.25	2.28
*SD*	0.85	0.15	0.14	0.54	0.84
Strictness					
*Mean*	2.08	1.32	2.56	2.76	2.07
*SD*	0.77	0.15	0.32	0.47	0.16

**Table 2:** MANOVA factorial (4^a^ × 2^b^) test for five dimensions of personality traits, satisfaction with life, and self-esteem.

Source of Variation	Λ	*F*	*df*_between_	*df*_error_
(A) Parenting Style	0.697	6.46 ***	21	1011.30
(B) Sex	0.912	4.48 ***	7	352.00
A × B	0.912	1.563	21	1011.30

*** *p* < 0.001.

**Table 3:** Univariate effects of parenting styles: means, standard deviations (in brackets), F values, probabilities of a type I error, and post-Bonferroni ^a^ procedure for the parenting style groups in five personality traits, satisfaction with life, and self-esteem.

	Parenting Style					
Source of Variation	Indulgent	Authoritative	Authoritarian	Neglectful	*F*_(3, 358)_	*p*
Five Factors IPIP						
*BFE*	3.31 (0.91) ^a^	3.00 (0.80)	2.85 (0.86) ^b^	2.87 (1.04)	3.03	0.035
*BFA*	4.31 (0.49) ^a^	3.97 (0.60) ^b^	3.75 (0.76) ^b2^	4.06 (0.59) ^1^	16.79	<0.001
*BFC*	3.38 (0.88) ^a^	3.29 (0.75)	2.99 (0.81) ^b^	3.14 (0.93)	3.48	0.026
*BFS*	3.03 (0.83) ^a^	2.96 (0.78) ^a^	2.64 (0.70) ^b^	2.55 (0.80) ^b^	9.08	<0.001
*BFI*	3.63 (0.71)	3.62 (0.71)	3.53 (0.79)	3.71 (0.89)	0.95	0.364
*Self-esteem*	3.24 (0.54) ^a^	3.19 (0.48) ^a^	2.68 (0.6) ^b^	2.7 (0.61) ^b^	2.67	<0.001
*Satisfaction with life*	3.22 (0.53) ^a^	3.20 (0.60) ^a^	2.56 (0.67) ^b^	2.51 (0.58) ^b^	26.25	<0.001

Note: Bonferroni test α = 0.05; ^a^ > ^b^, ^1^ > ^2^.

**Table 4:** Means, standard deviations (in brackets), and F values for sex in self-esteem, satisfaction with life, and the five personality traits.

Source of Variation	Sex		
	Boys	Girls	*F*_(1, 358)_
Five Factors IPIP			
*BFE*	2.95 (0.10)	3.03 (0.06)	0.443
*BFA*	3.86 (0.07)	4.12 (0.04)	11.434 ***
*BFC*	3.10 (0.09)	3.25 (0.06)	1.832
*BFS*	3.08 (0.08)	2.69 (0.05)	16.866 ***
*BFI*	3.62 (0.09)	3.64 (0.05)	0.039
*Self-esteem*	2.96 (0.06)	2.95 (0.04)	0.033
*Satisfaction with life*	2.82 (0.07)	2.89 (0.04)	1.003

Note: Bonferroni test α = 0.05; *** *p* < 0.001.

## Data Availability

The raw data supporting the conclusions of this article will be made available by the authors upon request.
